# Gemcitabine, Navelbine, and Doxorubicin as Treatment for Patients with Refractory or Relapsed T-Cell Lymphoma

**DOI:** 10.1155/2015/606752

**Published:** 2015-03-19

**Authors:** Zhengzi Qian, Zheng Song, Huilai Zhang, Xianhuo Wang, Jing Zhao, Huaqing Wang

**Affiliations:** Department of Lymphoma, Sino-US Center for Lymphoma and Leukemia, National Clinical Research Center of Cancer, Key Laboratory of Cancer Prevention and Therapy, Tianjin Medical University Cancer Institute and Hospital, Tiyuanbei, Huanhuxi Road, Hexi District, Tianjin 300060, China

## Abstract

T-cell lymphoma (TCL) is resistant to conventional chemotherapy. We retrospectively evaluated the therapeutic efficiency and toxicity of gemcitabine, navelbine, and doxorubicin (GND) in patients with refractory or relapsed TCL. From 2002 to 2012, 69 patients with refractory or relapsed TCL received GND treatment in our hospital. The treatment protocol comprised gemcitabine (800 mg/m^2^, group 1; 1000 mg/m^2^, group 2) on days 1 and 8, navelbine (25 mg/m^2^) on day 1, and doxorubicin (20 mg/m^2^) on day 1, repeated every 3 weeks. The overall response rate (ORR) was 65.2%. The median overall survival (OS) was 36 months. The 5-year estimated OS rate was 32.4%. The GND regimen was well tolerated. Subgroup analysis demonstrated that the ORR and CR for group 1 were similar. A longer median OS was observed for group 1. Significant difference in grades 3-4 toxicities was observed between groups 1 and 2 (*P* = 0.035). Our study indicated that gemcitabine (800 mg/m^2^) on days 1 and 8 every 21 days was favorable for pretreated TCL patients.

## 1. Introduction

T-cell lymphoma (TCL) belongs to a group of malignant, clonal hyperplastic diseases that is derived from T lymphocytes, and it is characterized by high heterogeneity, strong invasiveness, and a prominent association with Epstein-Barr virus and human T-lymphotropic virus type 1 infections as well as with specific chromosome translocations. The treatment outcomes of patients with B-cell lymphoma (BCL) have improved due to great advancements in chemotherapy combined with molecular targeted agents such as rituximab. However, due to its highly aggressive features, including local tumor invasiveness in early-stage disease, the outcomes of TCL patients are generally worse with poor long-term survival (5-year overall survival (OS): 20–30%) [1]. In addition, owing to resistance to conventional chemotherapeutic agents such as CHOP (cyclophosphamide, doxorubicin, vincristine, and prednisolone) or CHOP-like regimen, which is mediated by the expression of multidrug-resistance proteins, a substantial proportion of TCL patients develop refractory or relapsed disease. Although high-dose chemotherapy supported by autologous stem cell transplantation (ASCT) offers an advantage for some patients, the severe toxicities including cardiac and hematological adverse effects limit their widespread use. Even the introduction of novel drugs such as L-asparaginase cannot overcome the refractoriness completely. Therefore, additional trials and further studies are needed to develop safe and effective salvage chemotherapy regimens for patients with refractory or relapsed TCL.

Gemcitabine (2′,2′-difluoro-2′-deoxycytidine), which mainly acts on the synthesis phase of the cell cycle by inhibiting DNA synthesis, is a pyrimidine antimetabolite. It has been demonstrated that gemcitabine is one of the most effective agents when used either as a monotherapy agent or as part of a combination regimen for patients with relapsed or refractory Hodgkin lymphoma (HL) and non-Hodgkin lymphoma (NHL) [1–3]. Of particular importance, the National Comprehensive Cancer Network has incorporated this nucleoside metabolic inhibitor into its clinical practice guidelines.

Given the encouraging outcomes of previous studies, we investigated the effectiveness, safety, and toxicity of gemcitabine, navelbine, and doxorubicin (GND) combination chemotherapy in patients with refractory or relapsed TCL.

## 2. Patients and Methods

### 2.1. Patients

The subjects of this retrospective study are patients with refractory or relapsed TCL, who received GND treatment between January 2002 and December 2012 in the Tianjin Medical University Cancer Hospital. Patients were eligible according to the following criteria: histological with immunohistochemical diagnosis of TCL from professional pathologists according to the Revised European-American Lymphoma classification [4] and available pathological reports; complete blood counts showing white blood cell (WBC) counts ≥4 × 10^9^/L, platelet (PLT) counts ≥100 × 10^9^/L, neutrophil counts ≥1.5 × 10^9^/L, and hepatic and renal function tests demonstrating aspartate aminotransferase and alanine transaminase levels ≤35 U/L and serum creatinine ≤80 *μ*mol/L at the beginning of the treatment; no abnormalities with electrocardiography (ECG); refractory or relapsed after conventional therapeutic approaches including chemotherapy and/or radiotherapy; and accumulated dose of doxorubicin ≤ 350 mg/m^2^ during the previous treatment. Exclusion criteria included a history of hepatitis B, hepatitis C, human immunodeficiency virus, uncontrolled infection or significant cardiac dysfunction, or central nervous system lymphoma at the time of GND administration. We collected the following clinical characteristics of enrolled patients retrospectively: patient demographics, time until relapse, histopathologic subtypes, Eastern Cooperative Oncology Group performance status, extent of disease involvement, Ann Arbor stage, International Prognostic Index, serum *β*2 microglobulin (*β*2-MG) levels, lactate dehydrogenase (LDH) levels, previous treatment regimens, deadline of the follow-up examination, and cause of death.

### 2.2. Treatment Protocol

From our archived clinical records, we established a cohort of 69 patients who received 2–6 cycles (median, 4 cycles) of the GND regimen every 3 weeks. All drugs were diluted in normal saline solution and administered through the subclavian vein. The treatment protocol consisted of gemcitabine (800 mg/m^2^ or 1000 mg/m^2^) on days 1 and 8, navelbine (25 mg/m^2^) on days 1 and 8, and doxorubicin (20 mg/m^2^) on day 1. In addition, 17 patients received local radiotherapy (36 Gy) for lymphoma masses after the completion of chemotherapy. Prophylactic 5-HT3 receptor antagonist (ramosetron and granisetron) and dexamethasone were administered routinely 30 minutes before every cycle. All patients were required to undergo a routine examination including physical examination, standard blood counts, liver and kidney function tests, urine routine analysis, and ECG on day 1 of each cycle. If the results showed no marked abnormalities, the subsequent cycle of chemotherapy was continued. Otherwise, patients whose WBC counts were <4 × 10^9^/L and neutrophil counts were <1.5 × 10^9^/L received recombinant human granulocyte colony-stimulating factor (G-CSF) at a dose of 100 *μ*g/d and patients with PLT counts <100 × 10^9^/L received thrombopoietin (TPO) at the discretion of the treating physician, resulting in a treatment delay for 3–7 days.

### 2.3. Response Evaluation

All patients underwent a reevaluation with complete physical examination, laboratory tests, and previously positive radiographic examinations such as computed tomography (CT), magnetic resonance imaging, and positron emission tomography-CT imaging after every 2 cycles of the GND regimen. The tumor response was classified as complete remission (CR), unconfirmed complete remission (CRu), partial remission (PR), stable disease (SD), and progressive disease (PD), according to the International Workshop criteria for NHL [5]. The overall response rate (ORR) consists of CR, CRu, and PR. Adverse effects were also observed and graded from degree 1 to degree 4, according to the National Cancer Institute Common Terminology Criteria for Adverse Events v3.0. Overall survival (OS) was measured from the first day of GND treatment to the date of death due to any cause or the date of the last follow-up visit (30 June 2013).

### 2.4. Statistical Method

The SPSS software (Statistical Package for Social Science for Windows, version 17.0) for Windows was used for data analysis. Statistical significance was defined at *P* values < 0.05 by using a two-sided significance test. The survival rate was estimated and the survival curve was drawn simultaneously with the Kaplan-Meier method. Comparisons between response rates were performed by using the Chi-squared test (*χ* ^2^-test). The median OS is shown with 95% confidence interval (CI) limits and estimators for 1-, 3-, and 5-year OS were determined concomitantly. To compare the potential association between variables and prognosis, the log-rank test was performed. Variables showing *P* values < 0.05 in univariate analyses were candidates for multivariate analysis, which was performed by using the Cox proportional hazard regression model.

## 3. Results

### 3.1. Patient Characteristics

The clinical characteristics of 69 patients that were retrieved from clinical and pathological reports are summarized in Table 1. First, patients were stratified into 2 groups according to the different doses of gemcitabine, which were administered at either 800 mg/m^2^ in group 1 (*n* = 49) or 1000 mg/m^2^ in group 2 (*n* = 20). The time until recurrence from the initial diagnosis was calculated, and a cut-off of 12 months [6] was used to distinguish early relapse (48 patients (37 from group 1; 11 from group 2)) from late relapse (21 patients (12 from group 1; 9 from group 2)). Among all patients, peripheral TCL-unspecified (PTCL-U) is the most common histopathologic subtype (59.4%) followed by extranodal natural killer/T-cell lymphoma (33.3%), anaplastic large cell lymphoma (4.4%), and subcutaneous panniculitis-like TCL (2.9%). There was a male preponderance (42/69) in the cohort, and the median age was 59 years (range, 10–80 years). A majority of patients experienced B-symptoms and splenomegaly (53.6% and 58.0%, resp.). At baseline, 31 patients were classified as stages I-II and 38 patients were classified as stages III-IV. Remarkably, most patients showed elevated *β*2-MG levels (56.5%), LDH levels (66.7%), and most frequently elevated lymphocyte counts (75.4%). The previous chemotherapy treatments included CHOP or CHOP-like regimens (COP, CHOEP, ECHOP, and CHOPT), Hyper-CVAD (cyclophosphamide, vincristine, Adriamycin, and dexamethasone), DICE (dexamethasone, ifosfamide, carboplatin, and etoposide), and ICE (ifosfamide, carboplatin, and etoposide) with a median of 3 cycles (range, 2–6 cycles).

### 3.2. Response to GND

Table 2 demonstrates the clinical results of the two groups. Overall, objective responses to the GND regimen were obvious in 45 out of 69 evaluable patients with 20 patients achieving CR (29.0%) and 25 patients achieving PR (36.2%), resulting in an ORR of 65.2%. A total of 11 and 13 patients responded and developed SD (15.9%) or PD (18.9%), respectively. In addition, among 20 patients who achieved CR, 3 patients proceeded to receive ASCT and 5 patients received biotherapy. In subgroup analysis, the ORR was similar between patients from group 1 and group 2 (65.3% versus 65.0%, *P* = 0.981), although patients from group 1 achieved a higher CR rate than patients from group 2 (30.6% versus 25.0%, *P* = 0.641). Higher PR rates were observed in patients from group 2 versus group 1 (34.7% versus 40.0%, *P* = 0.677). There were no statistically significant response rate differences between the two different groups (by using *χ* ^2^-test).

### 3.3. Survival Analysis

**Figure 1:**
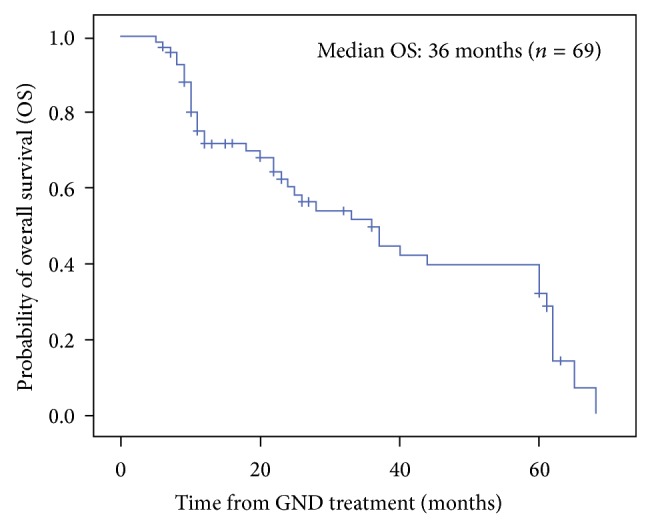
The Kaplan-Meier estimate of overall survival (OS) for all patients.

**Figure 2:**
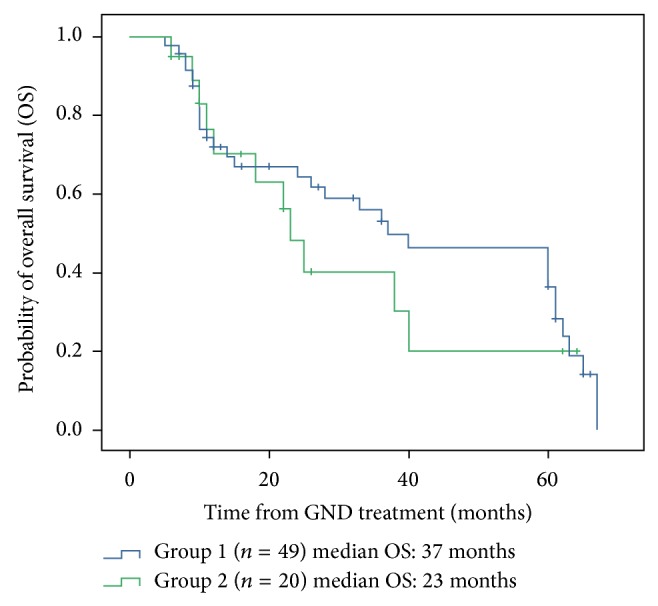
The Kaplan-Meier estimate of overall survival (OS) for groups 1 and 2.

At the cut-off date of the follow-up examination (30 June 2013), the median follow-up time was 3.5 years for all patients and 4 years for surviving patients (range, 0.5–11 years). The median OS was 36 months (range, 5–67 months; 95% CI: 25.314–46.686) among all patients. The median OS was higher for patients from group 1 compared to patients from group 2 (37 versus 23 months, resp.). According to the Kaplan-Meier analysis, the 1-, 3-, and 5-year estimated OS rates for the whole cohort were 71.7%, 47.3%, and 32.4%, respectively (Figure 1). Estimators for 1-year OS rates were similar between groups 1 and 2 (72.2% versus 70.3%, resp.). However, we observed significant differences for the 3- and 5-year OS rates between patients from groups 1 and 2 (53.1% versus 30.1% and 36.5% versus 20.1%, resp. (Figure 2)).

As shown in Table 1, univariate analysis identified 8 unfavorable prognostic factors for the 69 enrolled patients, including the time until recurrence (*P* = 0.021), B-symptoms (*P* = 0.014), bone marrow involvement (*P* = 0.000), splenomegaly (*P* = 0.010), disease stage (*P* = 0.004), lymphocyte counts (*P* = 0.005), *β*2-MG levels (*P* = 0.001), and LDH levels (*P* = 0.002). Moreover, multivariate Cox model analysis revealed that bone marrow involvement (*P* = 0.042; hazard ratio (HR): 3.816; 95% CI: 1.049–13.886), lymphocyte counts (*P* = 0.000; HR: 5.305; 95% CI: 2.100–13.403), and LDH levels (*P* = 0.018; HR: 2.538; 95% CI: 1.172–5.493) significantly influenced OS.

### 3.4. Treatment Toxicities

The GND regimen was well tolerated with grade 3 or greater treatment-emergent adverse events occurring in less than one-third of all responding patients. Unexpectedly, a significant difference in grade 3 to 4 toxicities was present between groups 1 and 2 (16.3% versus 40%, *P* = 0.035, by using *χ* ^2^-test). With regard to hematologic toxicities, which were more frequent relatively among all patients, grade 1 to 2 neutropenia or leukopenia was reported in 35 patients (50.7%), grade 1 to 2 anemia was noted in 23 patients (33.3%), and grade 1 to 2 thrombocytopenia was observed in 18 patients (26.1%). Grade 1 to 2 hematologic toxicities for group 2 patients were higher than those for group 1 patients.  Table 3 displays the specific proportions for the different groups. Although 21.7% of patients (group 1: 16.3%; group 2: 35.0%) developed grade 3 to 4 neutropenia or leukopenia, no grade 3 to 4 anemia or thrombocytopenia was observed. By using G-CSF and TPO, these hematological toxicities were easily manageable and mostly of short duration (≤1 week). Only 1 patient from group 2 had a neutropenia-associated pulmonary infection and recovered after anti-infective therapy. Nonhematological toxicities included nausea, emesis, fatigue, fever, headache, decreased appetite, constipation, and temporary dysfunction of the liver and kidney; most of these were mild and reversed spontaneously. No patients presented with severe pulmonary toxicity, catarrh, rash, dyspnea, anaphylaxis, edema, or peripheral nerve toxicity. Treatment related deaths did not occur. Other adverse effects included fever, headache, and temporary dysfunction of the liver and kidney.

## 4. Discussion

TCL encompasses a heterogeneous group of diseases, altogether accounting for less than 15% of all NHLs worldwide. It is known for its aggressive biological behavior, low response rate to initial treatment accompanied with a high recurrence rate, and poor prognosis even for stage I to II disease. Previously, many advances have been made in the treatment of TCL. Unfortunately, initiatives that just mirrored the therapies used for BCL have not achieved promising outcomes in TCL patients, especially in cases with relapsed or refractory disease. Because of the disappointing responses and serious toxicities, few options remain for therapeutic approaches incorporating novel agents such as alemtuzumab, bortezomib, or L-asparaginase containing regimes [7–9]. In addition, there is a paucity of data and consensus from phase III trials concerning the treatment of pretreated TCL patients.

Gemcitabine, a novel nucleoside analogue that is activated by deoxycytidine kinase (dCK), has shown promising results in solid tumors such as nonsmall cell lung cancer and in pancreatic and ovarian cancers [10–12]. Notably, recent studies showed that gemcitabine alone and/or gemcitabine containing chemotherapies were also efficient in the treatment of HL and NHL, including heavily pretreated lymphoma [1–3, 9]. In a phase II study of 44 pretreated patients with mycosis fungoides or cutaneous peripheral PTCL-U, this agent presented an attractive treatment option with a surprisingly high RR of 70.5% [13]. Furthermore, Marchi et al. reported RR of 75% with gemcitabine monotherapy in a phase II study of 32 previously untreated cutaneous TCL patients, with 22% of patients achieving CR [14]. Bergman et al. explored the possible mechanisms* in vitro* and found that gemcitabine acts against various human malignant cells with a multidrug resistance (MDR) phenotype by circumventing MDR [15]. MDR, associated with cross-resistance to some natural toxin-related compounds, is characterized by the overexpression of drug efflux pumps such as P-glycoprotein and MDR-associated proteins 1–3, which may be a result of increased dCK activity and reduced deoxycytidine deaminase activity [16]. Therefore, MDR cells often presented with accumulated gemcitabine metabolism and sensitivity. This mechanism was related to the incorporation of gemcitabine into DNA and RNA, which in turn led to DNA damage [15].

According to previous studies, the effectiveness of gemcitabine is demonstrated with satisfactory response rates and acceptable toxicities. However, there are very limited data available describing the efficacy and safety of gemcitabine combined with navelbine and specifically about doxorubicin as treatment for patients with refractory or relapsed TCL. In this report, we retrospectively analyzed a cohort of 69 patients with a range of pretreated TCL histology, who had received the gemcitabine-containing regimen, GND.

The ORR was 65.2%, including 29.0% of patients who achieved CR and a significant survival benefit (median OS: 36 months). Our observations are encouraging and comparable to other published salvage regimens such as ICE [17] and DHAP [18]. Even though those intensive regimens could achieve an ORR of 60–70% [17, 18], significant toxicities, especially serious complications related to myelosuppression, affected patients' survival. In contrast, mild bone marrow toxicity with GND was another significant advantage over other regimens, as only 15 patients (21.7%) developed grade 3 to 4 neutropenia or leukopenia. The incidence of grade 3 or 4 nonhematological toxicity was low, and severe pulmonary toxicity associated with gemcitabine [19] was not observed. In addition, these promising results were observed in a cohort of refractory or relapsed patients, many of which were characterized according to poor prognostic features such as early relapse [6], stages III-IV disease, elevated LDH and *β*2-MG levels, and elevated lymphocyte counts [20, 21].

The different outcomes may be due to the schedule or dose intensity of our study compared to historical reports. Grade 3 to 4 myelosuppression related toxicity as documented in the Royal Marsden Hospital experience [22] for CALGB 59804 was common (grade 3 to 4 neutropenia, 62% and 63%, separately) [3]. In addition, it is well established that navelbine and doxorubicin, which act on different parts of the cell cycle, play an important role in the management of malignant lymphomas, especially in the first-line treatment. Thus, the GND regimen did not contain alkylating agents such as ifosfamide and cyclophosphamide, which could increase the risk of secondary malignancies in patients with NHL [23].

In the further subgroups, in which gemcitabine was given at different doses, the OS and treatment-associated adverse events, particularly grade 3 to 4 toxicities (16.3% versus 40% in groups 1 and 2, resp., *P* = 0.035), were significantly different despite similar ORRs (65.3% versus 65% in groups 1 and 2, resp., *P* = 0.981). The outcome of our study indicates that gemcitabine at 800 mg/m^2^ on days 1 and 8 schedule repeated every 21 days was favorable for pretreated TCL patients.

## 5. Conclusion

In summary, our retrospective analysis showed that the GND treatment regimen was effective and well tolerated by patients with refractory or relapsed TCL. When interpreting the outcome of our study, the limited number of cases should be kept in mind. Therefore, further prospective investigations that involve a larger number of patients will be helpful to confirm the advantages of the GND regime and elucidate its clinical significance intensively.

## Figures and Tables

**Table 1:** Clinical characteristics and prognostic factors for overall survival (OS) of all patients.

Characteristics	Number of patients (%)			Univariate	Multivariate		
	Group 1	Group 2	Total	*P* value	*P* value	HR	95% CI
Total	49 (71%)	20 (29%)	69 (100%)				
Recurrent time				0.021			
Early relapse	37 (75.5%)	11 (55%)	48 (69.6%)				
Late relapse	12 (24.5%)	9 (45%)	21 (30.4%)				
Pathology							
PTCL-U	28 (57.1%)	13 (65%)	41 (59.4%)				
NK/T	18 (36.7%)	5 (25%)	23 (33.3%)				
Subcutaneous panniculitis-like	2 (4.1%)	0 (0%)	2 (2.9%)				
T-cell lymphoma							
ALCL	1 (2.1%)	2 (10%)	3 (4.4%)				
Sex							
Male	30 (61.2%)	12 (60%)	42 (60.9%)				
Female	19 (38.8%)	8 (40%)	27 (39.1%)				
Age, years							
Median (range)	50 (10–79)	58 (19–80)	59 (10–80)				
≤60	20 (40.8%)	9 (45%)	29 (42.0%)				
>60	29 (59.2%)	11 (55%)	40 (58.0%)				
B-symptoms				0.014			
Present	27 (55.1%)	10 (50%)	37 (53.6%)				
Absent	22 (44.9%)	10 (50%)	32 (46.4%)				
Marrow involvement				0.000	0.042	3.816	1.049–13.886
Present	9 (18.4%)	5 (25%)	14 (20.3%)				
Absent	40 (81.6%)	15 (75%)	55 (79.7%)				
Splenomegaly				0.010			
Present	29 (59.2%)	11 (55%)	40 (58.0%)				
Absent	20 (40.8%)	9 (45%)	29 (42.0%)				
ECOG performance status							
0-1	19 (38.8%)	8 (40%)	27 (39.1%)				
2	26 (53.1%)	11 (55%)	37 (53.6%)				
≥3	4 (8.1%)	1 (5%)	5 (7.3%)				
Stage				0.004			
I-II	24 (49.0%)	7 (35%)	31 (44.9%)				
III-IV	25 (51.0%)	13 (65%)	38 (55.1%)				
IPI							
0-1 (low-risk group)	19 (38.8%)	2 (10%)	21 (30.4%)				
2-3 (intermediate-risk group)	25 (51.0%)	14 (70%)	39 (56.5%)				
4-5 (high-risk group)	5 (10.2%)	4 (20%)	9 (13.1%)				
Lymphocyte counts				0.005	0.000	5.305	2.100–13.403
≥1 × 10^9^/L	36 (73.5%)	16 (80%)	52 (75.4%)				
<1 × 10^9^/L	13 (26.5%)	4 (20%)	17 (24.6%)				
*β*2-MG				0.001			
>Upper limit of normal	26 (53.1%)	13 (65%)	39 (56.5%)				
Normal	23 (46.9%)	7 (35%)	30 (43.5%)				
LDH				0.002	0.018	2.538	1.172–5.493
>Upper limit of normal	31 (63.3%)	15 (75%)	46 (66.7%)				
Normal	18 (36.7%)	5 (25%)	23 (33.3%)				
Previous therapeutic regimen							
Radiotherapy	9 (18.4%)	3 (15%)	12 (17.4%)				
Chemotherapy	29 (59.2%)	13 (65%)	42 (60.9%)				
Chemoradiotherapy	11 (22.4%)	4 (20%)	15 (21.7%)				

PTCL-U: peripheral T-cell lymphoma-unspecified, NK/T: extranodal natural killer/T-cell lymphoma, ALCL: anaplastic large cell lymphoma, ECOG: Eastern Cooperative Oncology Group, LDH: lactate dehydrogenase, *β*2-MG: serum *β*2 microglobulin, IPI: International Prognostic Index, HR: hazard ratio, and 95% CI: 95% confidence interval. B-symptoms include unexplained fever over 38°C (100.4°F) for 1-2 weeks, unintentional weight loss of >10% of normal body weight over a period of 6 months or less, and drenching sweats, especially at night. IPI scores were calculated by summing the number of risk factors (age > 60 years, stage III/IV, involved extranodal sites > 1, ECOG performance status > 1, and elevated LDH levels).

**Table 2:** The clinical results for the two groups.

Response	Number of patients (%)		
	Group 1 (*n* = 49)	Group 2 (*n* = 20)	Total (*n* = 69)

CR	15 (30.6%)	5 (25%)	20 (29.0%)
PR	17 (34.7%)	8 (40%)	25 (36.2%)
ORR (CR + PR)	32 (65.3%)	13 (65%)	45 (65.2%)
SD	8 (16.3%)	3 (15%)	11 (15.9%)
PD	9 (18.4%)	4 (20%)	13 (18.9%)

CR: complete response, PR: partial response, ORR: overall response rate, SD: stable disease, and PD: progressive disease.

**Table 3:** Treatment-emergent adverse events for the two groups.

Treatment toxicities	Number of patients (%)		
	Group 1 (*n* = 49)	Group 2 (*n* = 20)	Total (*n* = 69)

Grades 1-2			
Neutropenia or leukopenia	22 (44.9%)	13 (65%)	35 (50.7%)
Anemia	15 (30.6%)	8 (40%)	23 (33.3%)
Thrombocytopenia	11 (22.4%)	7 (35%)	18 (26.1%)
Infection	0	1 (5%)	1 (1.4%)
Nausea or emesis	25 (51.0%)	9 (45%)	34 (49.3%)
Fatigue	31 (63.3%)	13 (65%)	44 (63.8%)
Constipation	19 (38.8%)	10 (50%)	29 (42.2%)
Others	5 (10.2%)	2 (10%)	7 (10.1%)
Grades 3-4			
Hematologic toxicities	8 (16.3%)	7 (35%)	15 (21.7%)
Nonhematological toxicities	0	1 (5%)	1 (1.4%)

## References

[1] Zinzani P. L., Venturini F., Stefoni V. (2009). Gemcitabine as single agent in pretreated T-cell lymphoma patients: evaluation of the long-term outcome. *Annals of Oncology*.

[2] Bai B., Huang H.-Q., Cai Q.-Q. (2013). Promising long-term outcome of gemcitabine, vinorelbine, liposomal doxorubicin (GVD) in 14-day schedule as salvage regimen for patients with previously heavily treated Hodgkin's lymphoma and aggressive non-Hodgkin's lymphoma. *Medical Oncology*.

[3] Bartlett N. L., Niedzwiecki D., Johnson J. L. (2007). Gemcitabine, vinorelbine, and pegylated liposomal doxorubicin (GVD), a salvage regimen in relapsed Hodgkin's lymphoma: CALGB 59804. *Annals of Oncology*.

[4] Pileri S. A., Leoncini L., Falini B. (1995). Revised European-American lymphoma classification. *Current Opinion in Oncology*.

[5] Cheson B. D., Pfistner B., Juweid M. E. (2007). Revised response criteria for malignant lymphoma. *Journal of Clinical Oncology*.

[6] Guglielmi C., Gomez F., Philip T. (1998). Time to relapse has prognostic value in patients with aggressive lymphoma enrolled onto the parma trial. *Journal of Clinical Oncology*.

[7] Kim S. J., Kim K., Park Y. (2012). Dose modification of alemtuzumab in combination with dexamethasone, cytarabine, and cisplatin in patients with relapsed or refractory peripheral T-cell lymphoma: analysis of efficacy and toxicity. *Investigational New Drugs*.

[8] Lee J., Suh C., Kang H. J. (2008). Phase I study of proteasome inhibitor bortezomib plus CHOP in patients with advanced, aggressive T-cell or NK/T-cell lymphoma. *Annals of Oncology*.

[9] Ahn H. K., Kim S. J., Hwang D. W. (2013). Gemcitabine alone and/or containing chemotherapy is efficient in refractory or relapsed NK/T-cell lymphoma. *Investigational New Drugs*.

[10] Manegold C., Bergman B., Chemaissani A. (1997). Single-agent gemeitabine versus cisplatln-etoposlde: early results of a randomised phase II study in locally advanced or metastatic non-small-cell lung cancer. *Annals of Oncology*.

[11] Burris H. A., Moore M. J., Andersen J. (1997). Improvements in survival and clinical benefit with gemcitabine as first- line therapy for patients with advanced pancreas cancer: a randomized trial. *Journal of Clinical Oncology*.

[12] Rose P. G., Mossbruger K., Fusco N., Smrekar M., Eaton S., Rodriguez M. (2003). Gemcitabine reverses cisplatin resistance: demonstration of activity in platinum- and multidrug-resistant ovarian and peritoneal carcinoma. *Gynecologic Oncology*.

[13] Zinzani P. L., Baliva G., Magagnoli M. (2000). Gemcitabine treatment in pretreated cutaneous T-cell lymphoma: experience in 44 patients. *Journal of Clinical Oncology*.

[14] Marchi E., Alinari L., Tani M. (2005). Gemcitabine as frontline treatment for cutaneous T-cell lymphoma: phase II study of 32 patients. *Cancer*.

[15] Bergman A. M., Pinedo H. M., Talianidis I. (2003). Increased sensitivity to gemcitabine of P-glycoprotein and multidrug resistance-associated protein-overexpressing human cancer cell lines. *British Journal of Cancer*.

[16] Bergman A. M., Munch-Petersen B., Jensen P. B. (2001). Collateral sensitivity to gemcitabine (2′,2′-difluorodeoxycytidine) and cytosine arabinoside of daunorubicin- and VM-26-resistant variants of human small cell lung cancer cell lines. *Biochemical Pharmacology*.

[17] Moskowitz C. H., Bertino J. R., Glassman J. R. (1999). Ifosfamide, carboplatin, and etoposide: a highly effective cytoreduction and peripheral-blood progenitor-cell mobilization regimen for transplant-eligible patients with non-Hodgkin's lymphoma. *Journal of Clinical Oncology*.

[18] Velasquez W. S., Cabanillas F., Salvador P. (1988). Effective salvagae therapy for lymphoma with cisplatin in combination with high-dose ara-C and dexamethasone (DHAP). *Blood*.

[19] Barlési F., Doddoli C., Gimenez C., Greillier L., Lima G., Kleisbauer J.-P. (2003). Acute pulmonary toxicity due to gemcitabine: a role for asbestos exposure?. *Revue des Maladies Respiratoires*.

[20] Castillo J. J., Morales D., Quinones P., Cotrina E., Desposorio C., Beltran B. (2010). Lymphopenia as a prognostic factor in patients with peripheral T-cell lymphoma, unspecified. *Leukemia and Lymphoma*.

[21] Escalón M. P., Liu N. S., Yang Y. (2005). Prognostic factors and treatment of patients with T-cell non-Hodgkin lymphoma: the M. D. Anderson Cancer Center experience. *Cancer*.

[22] Arkenau H.-T., Chong G., Cunningham D. (2007). Gemcitabine, cisplatin and methylprednisolone for the treatment of patients with peripheral T-cell lymphoma: the Royal Marsden Hospital experience. *Haematologica*.

[23] Xu Y., Wang H., Zhou S. (2013). Risk of second malignant neoplasms after cyclophosphamide-based chemotherapy with or without radiotherapy for non-Hodgkin lymphoma. *Leukemia and Lymphoma*.

